# Minor Impact of A258D Mutation on Biochemical and Enzymatic Properties of *Leishmania infantum* GDP-Mannose Pyrophosphorylase

**DOI:** 10.3390/microorganisms10020231

**Published:** 2022-01-21

**Authors:** Wei Mao, Noureddine Lazar, Herman van Tilbeurgh, Philippe M. Loiseau, Sébastien Pomel

**Affiliations:** 1 Université Paris-Saclay, CNRS, BioCIS, 92290 Châtenay-Malabry, France; maowei@hotmail.fr (W.M.); philippe.loiseau@universite-paris-saclay.fr (P.M.L.); 2 Université Paris-Saclay, CEA, CNRS, Institute for Integrative Biology of the Cell (I2BC), 91198 Gif-sur-Yvette, France; noureddine.lazar@i2bc.paris-saclay.fr (N.L.); herman.van-tilbeurgh@i2bc.paris-saclay.fr (H.v.T.)

**Keywords:** GDP-mannose pyrophosphorylase, *Leishmania infantum*, enzyme properties

## Abstract

Background: Leishmaniasis, a vector-borne disease caused by the protozoan parasite from the genus *Leishmania*, is endemic to tropical and subtropical areas. Few treatments are available against leishmaniasis, with all presenting issues of toxicity, resistance, and/or cost. In this context, the development of new antileishmanial drugs is urgently needed. GDP-mannose pyrophosphorylase (GDP-MP), an enzyme involved in the mannosylation pathway, has been described to constitute an attractive therapeutic target for the development of specific antileishmanial agents. Methods: In this work, we produced, purified, and analyzed the enzymatic properties of the recombinant *L. infantum* GDP-MP (*Li*GDP-MP), a single leishmanial GDP-MP that presents mutation of an aspartate instead of an alanine at position 258, which is also the single residue difference with the homolog in *L. donovani*: *Ld*GDP-MP. Results: The purified *Li*GDP-MP displayed high substrate and cofactor specificities, a sequential random mechanism of reaction, and the following kinetic constants: V*_m_* at 0.6 µM·min^−1^, K*_m_* from 15–18 µM, k*_cat_* from 12.5–13 min^−1^, and k*_cat_*/K*_m_* at around 0.8 min^−1^µM^−1^. Conclusions: These results show that *Li*GDP-MP has similar biochemical and enzymatic properties to *Ld*GDP-MP. Further studies are needed to determine the advantage for *L. infantum* of the A258D residue change in GDP-MP.

## 1. Introduction

Leishmaniases are a complex of neglected tropical and sub-tropical diseases caused by the protozoan parasite from the genus *Leishmania* and transmitted by phlebotomine sandflies. During its life cycle, the parasite alternates from a flagellate mobile form in the insect vector, called a promastigote, to an intracellular form in mammalian cells, called an amastigote. Three main forms of this parasitosis can be encountered: (i) cutaneous (CL), which causes skin sores and ulcers and can be self-healing; (ii) muco-cutaneous (MCL), which causes disfiguring skin lesions; and (iii) visceral (VL), which is lethal without treatment. Currently, 50,000 to 90,000 new cases of VL and 600,000 to 1 million new cases of CL are estimated to occur annually worldwide [1]. Depending on the *Leishmania* species, the parasites display a preferential cutaneous or visceral tropism, and are differentially distributed over the world. For instance, CL can be caused by *L. major* in North Africa and *L. mexicana* in South America, MCL by *L. braziliensis* in South America, and VL by *L. donovani* in East Africa and India. Human VL cases are mostly caused by two *Leishmania* species worldwide: *L. donovani* and *L. infantum*. These latter *Leishmania* species are phylogenetically closely related, as they are in the same *L. donovani* complex [2]. However, *L. infantum* causes zoonotic VL as its reservoir is the dog while *L. donovani* is anthroponotic [3]. Moreover, canine leishmaniasis (CanL) is endemic to the Mediterranean Basin, especially in Southern Europe, where an estimated 2.5 million dogs are currently infected by *L. infantum* [4,5]. The control of leishmaniasis mainly relies on chemotherapy despite limitations of toxicity and drug resistance [6]. Therefore, the development of new antileishmanial drugs is currently crucial to circumvent these drawbacks and to diversify the drug arsenal. In this way, a strategy consisting of conceiving new antileishmanial agents specifically directed against a biochemical target in the parasite appears to be promising.

The GDP-mannose pyrophosphorylase (GDP-MP) is a ubiquitous glycosylation enzyme catalyzing the formation of GDP-mannose from mannose-1-phosphate (Man-1-P) and GTP [7]. In *Leishmania*, this nucleotide-sugar is an activated form of mannose used to biosynthesize a wide range of glycoconjugates, such as LipoPhosGhoglycan (LPG), ProteoPhosphoGlycan (PPG), or GlycosylPhosphatidylInositol (GPI), which are essential for host–cell recognition [8]. A knockout of the gene encoding for GDP-MP in *L. mexicana* led to perturbed morphology and cytokinesis and to retarded growth in the promastigote form, and to a lack of development and rapid elimination of the amastigote form within the macrophage [9,10]. Moreover, these mutant parasites are rapidly cleared in infected mice, showing that GDP-MP is essential for amastigote survival both in vitro and in vivo [9,10]. Moreover, several differences have been identified in the catalytic site of leishmanial GDP-MP in comparison with the human counterpart [11,12], making this enzyme an attractive therapeutic target to develop new specific antileishmanial agents [13]. Furthermore, some studies highlight a possible relationship between GDP-MP and drug resistance [14,15].

Recombinant GDP-MPs from two geographically distant *Leishmania* species, *L. donovani* (*Ld*GDP-MP) and *L. mexicana* (*Lm*GDP-MP), and from human (*h*GDP-MP) have been previously expressed, produced, and purified, and their enzymatic properties have been characterized [16,17]. In the present work, we purified a third leishmanial GDP-MP, called *Li*GDP-MP, from the species *L. infantum*, presenting a unique amino acid difference with *Ld*GDP-MP. Following the determination of the optimal parameters of enzymatic reaction (temperature, pH, substrate and cofactor concentrations), we characterized the substrate and cofactor specificities, kinetic constants, and the mechanism of reaction of *Li*GDP-MP and compared them with those of *Ld*GDP-MP and *h*GDP-MP.

## 2. Materials and Methods

### 2.1. Chemicals and Media

All chemicals and solvents were purchased from Sigma-Aldrich (Saint-Quentin-Fallavier, France), except for GTP, malachite green, and Na-pyrophosphate, which were purchased from Fisher Scientific (Illkirch, France).

Dulbecco’s Modified Eagle’s Medium, Luria-Bertani (LB) broth medium and penicillin-streptomycin were purchased from Fisher Scientific (Illkirch, France).

### 2.2. Identification of Leishmanial GDP-MP Sequences

The leishmanial GDP-MP sequences were identified by BLASTP analysis (https://blast.ncbi.nlm.nih.gov/Blast.cgi?PAGE=Proteins; accessed on 16 January 2022) [18] using *Li*GDP-MP from *L. infantum* strain JPCM5 (MCAN/ES/98/LLM-877; accession number: CAM68115.1), *Ld*GDP-MP from *L. donovani* strain HU3 (MHOM/ET/67/HU3; accession number: CAC5430144.1), or any leishmanial GDP-MP further identified from *L. braziliensis* (strain MHOM/BR/75/M2904; accession number: CAM36519.1), *L. guyanensis* (strain MHOM/BR/75/M4147; accession number: CCM15710.1), *L. panamensis* (strain MHOM/PA/94/PSC-1; accession number: AIN98464.1), *L. mexicana* (strain MNYC/BZ/62/M379; accession number: CAC27419.1) or *L. major* (strain MHOM/IL/81/Friedlin; accession number: CAJ03868.1) as a query. All these leishmanial GDP-MP sequences, besides *h*GDP-MP (β2 subunit; accession number: NP_068806.1) and *T. maritima* GDP-MP (accession number: 2X5S_A), which were previously crystallized, were further aligned using Clustal Omega [19,20].

### 2.3. DNA Extraction, PCR and Cloning Strategy

The genomic DNA (gDNA) from *L. infantum* JPCM5 strain (MCAN/ES/98/LLM-877) was extracted by DNAzol reagent according to the manufacturer’s instructions (Fisher Scientific, Illkirch, France). The Li*GDP-MP* gene was amplified from *L. infantum* gDNA using the following oligonucleotide primers: 5′-AACCATATGTCTGCATCCGATGGCC-3′ (forward-NdeI) and 5′-GATCTCGAGCATGATGATCCCAGGC-3′ (reverse-XhoI). The PCR conditions were the same as previously described by Mao and collaborators [16] to amplify the gene encoding for *Leishmania donovani* GDP-MP (*Ld*GDP-MP). Briefly, the PCR mix contained 1 ng of DNA, 1 mM MgCl_2_, 0.4 mM dNTPs, 2 pmol/µL of each primer, and 1 U of Hot Diamond Taq (Eurogentec, Liege, Belgium). The PCR was performed with (1) denaturation for 3 min at 95 °C, (2) 30 cycles of denaturation for 30 s at 95 °C annealing at 54 °C for 30 s elongation at 72 °C for 90 s, and (3) extension for 15 min at 72 °C.

The amplified fragment was purified on 1% agarose gel using a Qiaquick gel extraction kit (Qiagen, Courtaboeuf, France) and double digested with NdeI and XhoI (New England Biolabs, Evry, France). This fragment was ligated into a NdeI/XhoI digested pET-21b(+) expression vector (Merck, Fontenay-sous-Bois, France), which encodes for a His6 tag fused at the C-terminal end of the protein of interest. The product of ligation was transformed into the DH5α *E. coli* strain following the recommendations of the manufacturer (Fisher Scientific, Illkirch, France). The positive clones were selected with ampicillin antibiotic and the plasmids were extracted using a QIAprep Spin Miniprep kit (Qiagen, Courtaboeuf, France). The sequence of three positive clones was verified on both strands (Eurofins, Nantes, France), prior to *E. coli* BL21(DE3) (Fisher Scientific, Illkirch, France) transformation for protein expression.

All the following steps concerning the production, purification, and enzymatic assays of *Li*GDP-MP (Section 2.4, Section 2.5 and Section 2.6) were performed at the same time as a previously published study [16], side-by-side with *Ld*GDP-MP and *h*GDP-MP, allowing the avoidance of any technical variation and comparison of the biochemical and enzymatic properties between these GDP-MPs.

### 2.4. Production of Recombinant LiGDP-MP

The experimental conditions for the production of recombinant *Li*GDP-MP were identical to the conditions for the production of recombinant *Ld*GDP-MP previously described by Mao and collaborators [16]. Briefly, after an overnight preculture at 37 °C under rotation at 200 rpm in LB broth media supplemented with 100 µg/mL ampicillin, a 1:100 dilution of *E. coli* BL21(DE3) was inoculated in 800 mL of LB supplemented with 100 µg/mL ampicillin and cultured at 37 °C under rotation at 200 rpm until the OD_600nm_ reached 0.6. Protein expression was induced by adding IPTG (IsoPropyl β-D-1-ThioGalactopyranoside) to a final concentration of 0.1 mM and cultures were further incubated at 30 °C under rotation at 200 rpm for 4 h. The cells were harvested by centrifugation at 3300× *g* for 30 min at 8 °C and the pellet was suspended in 40 mL of lysis buffer containing 50 mM Tris-HCl pH 7.5, 200 mM NaCl, 0.1% Triton X-100 (*v*/*v*), 100 µM PMSF (PhenylMethylSulfonyl Fluoride), and 400 µg/mL lysozyme. The protein extract was then sonicated 4 times for 45 s on ice at an amplitude at 40 and duty cycle of 90 (Branson Sonifier 250; Fisher Scientific, Illkirch, France) and further centrifugated at 20,000× *g* for 30 min at 8 °C. The supernatant was collected and used for purification.

### 2.5. Purification of Recombinant LiGDP-MP

The supernatant prepared in Section 2.4 was first loaded on an affinity chromatography column containing Ni-nitrilotriacetic acid (Ni-NTA) agarose with immobilized nickel ion (Ni-NTA agarose, Qiagen; Courtaboeuf, France), pre-equilibrated with 50 mM Tris pH 7.5, 200 mM NaCl, 1 mM DTT (DiThioTreitol; buffer A). After a wash with buffer A, which contained 20 mM imidazole, the protein was eluted in buffer A, which contained increasing concentrations of imidazole, from 100 to 400 mM. Each eluted fraction was analyzed by 15% acrylamide SDS-PAGE (Sodium Dodecyl Sulfate Polyacrylamide Gel Electrophoresis) followed by Coomassie blue staining (0.1% Coomassie brilliant blue R-250 in 50% methanol, 10% acetic acid). The fractions containing the uppermost quantity of the protein of interest with the lowest amount of protein contaminants were then selected. The fractions containing *Li*GDP-MP were concentrated by an Amicon Ultra 5000 MWCO membrane tube (Merck, Fontenay-sous-Bois, France) and finally loaded on a Size Exclusion Chromatography (SEC) Superdex^TM^ 200 10/300 GL column (GE Healthcare, Buc, France), pre-equilibrated with 50 mM Tris-HCl pH 7.5, 1 M NaCl, 1 mM DTT, 10% glycerol. This column was connected to an NGC Chromatography Systems (Biorad, Marnes-La-Coquette, France). The SEC purification result was analyzed by SDS-PAGE followed by Coomassie blue staining, and the fraction containing *Li*GDP-MP was collected and concentrated at 12.8 mg/mL by an Amicon Ultra 5000 MWCO membrane tube (Merck, Fontenay-sous-Bois, France) for further enzymatic assays.

### 2.6. Enzymatic Assays

GDP-MP catalyzes the following reaction:

Man-1-P + GTP ⇆ GDP-Mannose + Pyrophosphate

The optimal condition assays of *Li*GDP-MP were determined by varying the conditions of a single parameter (time of reaction, enzyme concentration, substrate, cofactor, temperature, or pH) while maintaining optimal conditions for the other parameters, as previously described [16]. Optimization of the *Li*GDP-MP enzymatic assays was performed in triplicates in three independent experiments.

Briefly, the optimal *Li*GDP-MP enzymatic assays were performed at 37 °C for 20 min in a medium containing 50 mM Tris-HCl pH 7.5, 100 µM Man-1-P, 100 µM GTP, 5 mM MgCl_2_, 2 ng/µL *Li*GDP-MP (Supplementary Table S1), supplemented with 1 mM DTT. Inorganic pyrophosphatase, which hydrolyzes the inorganic pyrophosphates generated by the GDP-MPs in inorganic phosphates, was added at 0.1 U/mL (Sigma-Aldrich, Saint-Quentin-Fallavier, France). The reaction was initiated by the addition of GDP-MP and was stopped after adding 100 µL of revelation buffer containing malachite green (0.03% *w*/*v*), ammonium molybdate (0.2% *w*/*v*), and Triton X-100 (0.05% *v*/*v*) in HCl (0.7 M) for 5 min at 37 °C. The quantity of inorganic phosphate generated, representing GDP-MP activity, was determined by measuring OD_650nm_ (Lab systems Multiskan MS; Fisher Scientific, Illkirch, France). The molar extinction coefficient ε of the end product detected was calculated at 27,207 M^−1^cm^−1^ [16]. The kinetic constants K*_m_*, V*_m_*, and k*_cat_* were determined by nonlinear regression using GraphPad Prism 6.0 (GraphPad software company, San Diego, CA, USA).

## 3. Results

**Figure 1:**
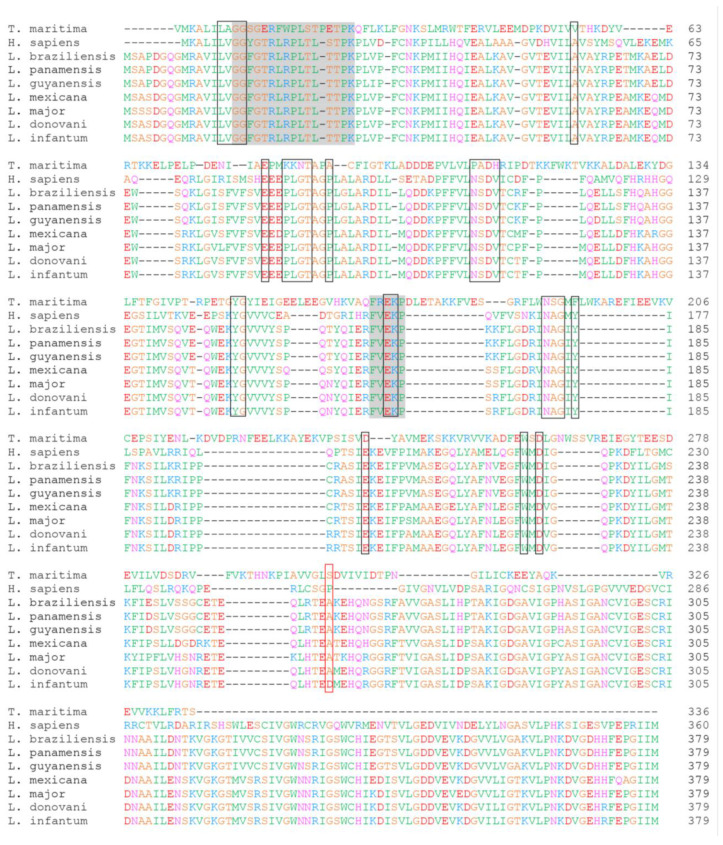
Sequence alignment of *T. maritima*, human, and leishmanial GDP-MPs. The consensus pyrophosphorylase signature sequence GXGXRLXPLX_5_PK and the GDP-MP active site motif F(V)EKP [11,19] are highlighted in grey. Black boxes represent the amino acids involved in GDP-mannose binding in *T. maritima* and human GDP-MPs identified from their crystal or cryo-EM structures previously determined, with the PDB (Protein Data Bank) codes 2X5Z and 7D72, respectively [19,20]. The red box, situated outside the active site, highlights the mutation A258D between *Li*GDP-MP and other leishmanial GDP-MPs. Sequence accession numbers of GDP-MPs from *T. maritima*, *H. sapiens* (*h*GDP-MP), *L. braziliensis*, *L. panamensis*, *L. guyanensis*, *L. mexicana*, *L. major*, *L. donovani* (*Ld*GDP-MP), and *L. infantum* (*Li*GDP-MP) are 2X5S_A, NP_068806.1, CAM36519.1, AIN98464.1, CCM15710.1, CAC27419.1, CAJ03868.1, CAC5430144.1, and CAM68115.1, respectively.

A sequence alignment study of 7 leishmanial GDP-MPs gathered by BLASTP analysis revealed that both *Ld*GDP-MP and *Li*GDP-MP display a high identity of 99.74%, with a difference of only one amino acid at position 258, from an alanine (A) in *Ld*GDP-MP to an aspartate (D) in *Li*GDP-MP (A258D; Figure 1). More importantly, among all the leishmanial GDP-MPs collected, *Li*GDP-MP was the single protein presenting an aspartate at position 258 (Figure 1), whereas the others, as for *Ld*GDP-MP, displayed an alanine at this position. Although this amino acid position is not involved in substrate binding and is outside the enzyme catalytic site, based on the GDP-MP crystal structures and molecular models previously described (Figure 1) [11,12,21,22], this residue difference is a major change, as aspartate is charged and not alanine. Therefore, in the present work, we purified *Li*GDP-MP in order to determine the effect of this residue substitution on the enzyme properties and to further characterize the similarities and specificities between human and leishmanial GDP-MPs.

**Figure 2:**
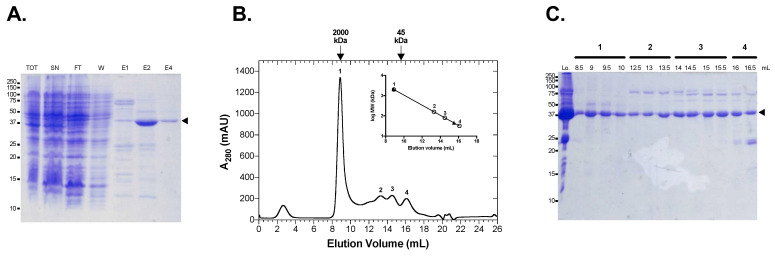
Purification of *Li*GDP-MP. (**A**) Coomassie blue-stained SDS-PAGE of *Li*GDP-MP purified from the Ni-NTA column. TOT: total; SN: Supernatant; FT: Flow Through; W: Wash; E1, E2 and E4: elution fraction at 100, 200, and 400 mM imidazole, respectively. The arrowhead shows the protein of interest. The protein ladder on the left is indicated in kDa. (**B**) Size exclusion chromatography profile of *Li*GDP-MP. In the inset, the filled triangles and open circles represent standard proteins and the peaks of the protein of interest, respectively. mAU = milli-Absorbance Unit. MW = Molecular Weight. (**C**) Coomassie blue-stained SDS-PAGE of the fraction volumes of peaks 1 (8.5–10 mL), 2 (12.5–13.5 mL), 3 (14–15.5 mL), and 4 (16–16.5 mL). Lo is the loading control. The arrowhead shows the protein of interest. The protein ladder on the left is indicated in kDa.

Following cloning of the Li*GDP-MP* gene into a bacterial expression vector, the recombinant His-6-tagged enzyme was produced and further purified using nickel affinity chromatography followed by size exclusion chromatography (Figure 2A). The gel filtration profile showed 4 peaks: one major peak at 9 mL followed by three smaller peaks at 13.5, 15, and 16 mL (Figure 2B). The analysis in SDS-PAGE of the fractions from peaks 1 to 4 revealed a major band at 42 kDa corresponding to the predicted molecular weight of *Li*GDP-MP and displaying 73% sequence coverage with *Li*GDP-MP in mass spectrometry, with the presence of a D residue at position 258 as expected (Figure 2C and Supplementary Figure S1). The estimated molecular weight of peak 1 was very high, at around 2000 kDa, with negligible enzyme activity, indicating that *Li*GDP-MP acquired an aggregated soluble form in these fractions (Figure 2B). However, the elution volumes of peaks 2, 3, and 4 approximately corresponded to the molecular weights of the hexameric, trimeric, and monomeric forms of the enzyme. All fractions of these three peaks displayed major and comparable enzyme activities and were therefore pooled for further enzymatic assays, as previously described for *Ld*GDP-MP [16].

**Figure 3:**
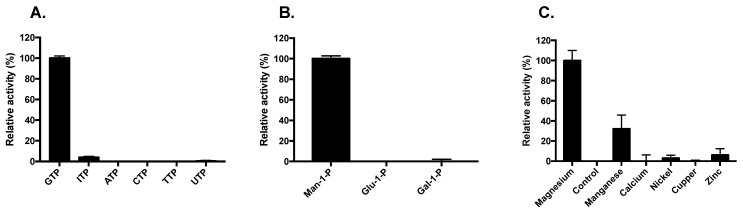
Determination of *Li*GDP-MP substrate and cofactor specificities. The NTPs GTP, ATP, CTP, TTP, UTP, and ITP (**A**), and sugar monophosphates Man-1-P, Glu-1-P, and Gal-1-P (**B**) were used at 100 µM. All cofactors Mg^2+^, Mn^2+^, Ca^2+^, Ni^2+^, Cu^2+^, and Zn^2+^ (**C**) were used at 5 mM. In each graph, enzyme activities were calculated relative to the maximal activity obtained and are expressed as a percent. The results correspond to the mean of three independent experiments ± SD.

The best reaction conditions of *Li*GDP-MP were obtained at 37 °C, pH 7.6, with a 30 min reaction time and an enzyme concentration of 2 ng/µL (Supplementary Figures S2–S4 and Table S1). Since no significant differences were obtained with a 20 min reaction time, as previously described for other leishmanial GDP-MPs and *h*GDP-MP [16], all further enzymatic reactions were performed with a reaction time of 20 min. The analysis of Michaelis–Menten plots allowed determination of the optimal concentrations of both substrates, Man-1-P and GTP, at 100 µM (Supplementary Figure S4D,E). Maximal *Li*GDP-MP activity was obtained with 5 mM Mg^2+^, and no activity was observed in the absence of this cofactor (Supplementary Figure S4C and Figure 3C). Furthermore, no GDP-MP activity was detected in the presence of other divalent ions, such as Ca^2+^, Ni^2+^, Cu^2+^, or Zn^2+^, except for Mn^2+^, which exhibited 32% of the activity compared to Mg^2+^ (Figure 3C). In addition, *Li*GDP-MP was inactive in the presence of any substrate analogue, either a nucleotide triphosphate distinct from GTP, or a sugar monophosphate other than Man-1-P (Figure 3A,B).

Following the characterization of the optimal conditions for the *Li*GDP-MP enzymatic reaction (Supplementary Table S1), we determined the kinetic constants of the enzyme for both substrates, GTP and Man-1-P, using Lineweaver–Burk double reciprocal plots (Supplementary Figure S5 and Table 1). Each kinetic constant was similar for GTP and Man-1-P: for both substrates, *Li*GDP-MP displayed a maximal velocity (V*_m_*) at 0.6 µM·min^−1^, K*_m_* between 15 and 18 µM, a turnover rate (k*_cat_*) between 12.5 and 13 min^−1^, and a catalytic efficiency (k*_cat_*/K*_m_*) of around 0.8 min^−1^µM^−1^ (Table 1).

**Figure 4:**
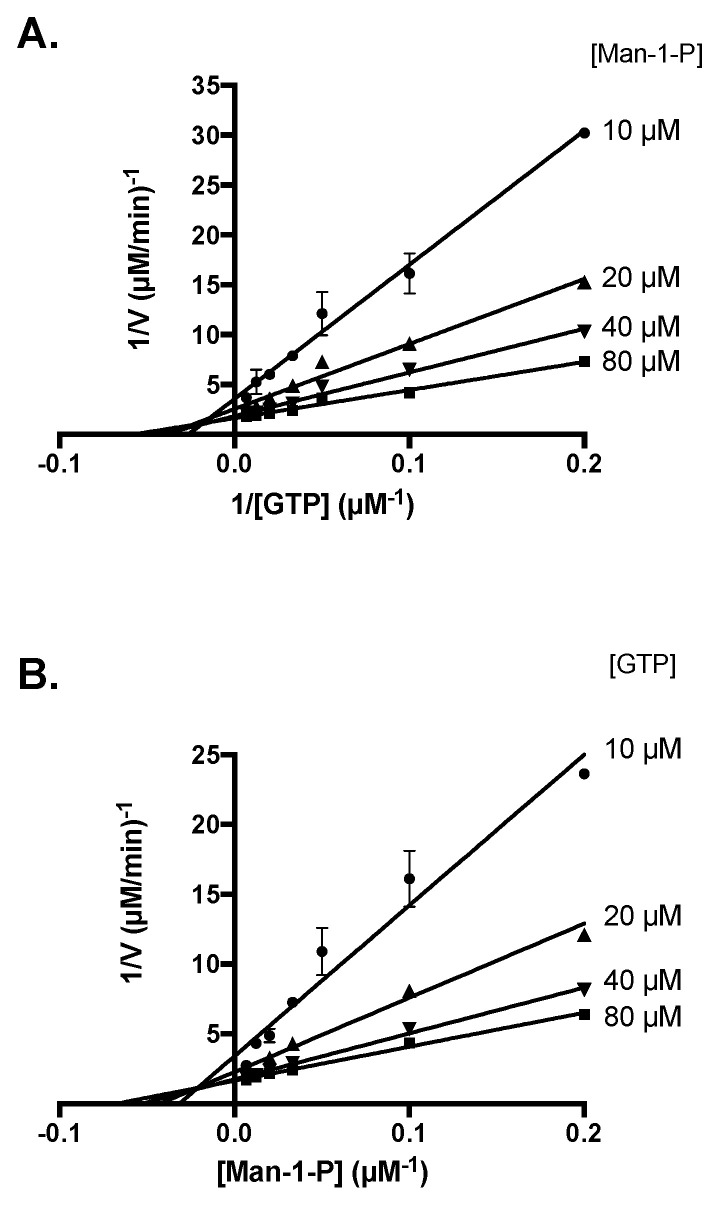
Determination of the *Li*GDP-MP mechanism of reaction. (**A**) Lineweaver–Burk plots 1/V = f(1/[GTP]). Constant concentrations of Man-1-P were held at 10, 20, 40, and 80 µM. (**B**) Lineweaver–Burk plots 1/V = f(1/[Man-1-P]). Constant concentrations of GTP were held at 10, 20, 40, and 80 µM. The results expressed correspond to the mean of three independent experiments ± SD.

In order to determine the kinetics of substrate binding, the mechanism of reaction of *Li*GDP-MP was then further investigated by measuring the enzyme activity using a range of GTP concentrations and fixed Man-1-P concentrations (Figure 4A), and vice versa (Figure 4B). Both double inverse plots intersect on the left of the 1/y axis. These results are therefore consistent with the formation of a ternary complex, composed of the enzyme, GTP, and Man-1-P, with a sequential mechanism of substrate binding [23]. Moreover, the intersection of both double plots above the 1/x axis is in favor of a sequential random mechanism, where the fixation of one substrate is favored by the previous fixation of the other.

## 4. Discussion

In the present work, the GDP-MP from *L. infantum* was produced in *Escherichia coli* and further purified in order to compare its biochemical and enzymatic properties with other recombinant GDP-MPs previously purified [16]. Two chromatography columns were necessary to purify *Li*GDP-MP, as previously described for *Ld*GDP-MP [16]: nickel affinity chromatography and size exclusion chromatography (SEC). This latter purification step showed that *Li*GDP-MP was purified in different oligomerization states: one at a very high molecular weight containing presumably soluble aggregates, and three others matching the hexamer, trimer, and monomer forms of the enzyme. Leishmanial GDP-MPs have previously been described to hexamerize in conditions with a high enzyme concentration, and to dissociate in trimers or monomers in low ionic conditions [16,24]. Depending on their quaternary structure stability, a mixture of hexameric, trimeric, and monomeric forms could be observed for some leishmanial GDP-MPs, such as *Ld*GDP-MP [16]. In mammals, native GDP-MP is a complex of 450 kDa composed of subunits α and β, where only β presents enzyme activity and α has a regulatory role [25,26]. Especially, native human GDP-MP is composed of four α subunits and eight β, with two sets of four subunits organized in two dimers of dimers, and single mutations in either α or β subunits alter the overall complex stability and enzyme activity [22]. Likewise, in other NTP-transferases, such as UDP-glucose pyrophosphorylase, single amino acid replacements, either in residues at the C-terminal end involved in enzyme oligomerization or in the active site, could alter the enzyme quaternary structure and/or activity [27,28]. However, in *Li*GDP-MP, the single mutation A258D, which is outside of the catalytic site at the C-terminal end, did not alter the enzyme quaternary structure’s stability, as the SEC profile was similar to the one previously obtained with *Ld*GDP-MP [16]. Nevertheless, this single mutation could affect protein interactions, and the enzyme location within the parasite, and further experiments would be needed to analyze in detail GDP-MP’s implications at the cellular level.

The optimal reaction conditions were identical between *Li*GDP-MP and *Ld*GDP-MP for all parameters measured: time of reaction (30 min), temperature (37 °C), pH (7.6), substrate (100 µM), enzyme (2 ng/µL), and Mg^2+^ concentrations (5 mM; Supplementary Table S1). Moreover, the enzyme *Li*GDP-MP displayed 32% of the activity with the cofactor Mn^2+^ in comparison with Mg^2+^, but no enzyme activity was observed with any substrate analogue. Likewise, *Ld*GDP-MP was not active in the presence of any GTP or Man-1-P analogue and displayed 33% of the activity in the presence of Mn^2+^ in comparison to Mg^2+^ [16]. These results show that, as for *Ld*GDP-MP, *Li*GDP-MP is highly specific to the substrates Man-1-P and GTP and to the cofactor Mg^2+^, besides moderate activity with Mn^2+^. A high substrate specificity was also previously described for bacterial and human GDP-MPs [16,29]. In *T. brucei*, GDP-MP was also described to be very specific to GTP and Man-1-P, apart from a weak affinity observed for ATP, with a K*_m_* value of 290 µM [30]. Moreover, in line with our results, bacterial GDP-MP has been described to display approximately 50% of the activity in the presence of Mn^2+^ [31]. Furthermore, in contrast to *Li*GDP-MP or *Ld*GDP-MP, *h*GDP-MP displayed a slightly acidic pH optimum (pH 6.5), required a low Mg^2+^ concentration at 1 mM, did not show any activity in the presence of Mn^2+^, and displayed 24% of the activity in the presence of the nucleotide ITP in comparison to GTP (Supplementary Table S1) [16].

Concerning the kinetic constants, *Li*GDP-MP displayed K*_m_* of 15 and 18 µM for Man-1-P and GTP, respectively, in the same range as *Ld*GDP-MP, while *h*GDP-MP presented a noticeably higher affinity to both substrates with K*_m_* values of ≈7 µM [16]. However, these differences seem to be minor since K*_m_* values for *h*GDP-MP have recently been determined at 11 to 12 µM for both substrates [22]. Therefore, our results in the present work, in line with data previously published [16,22,25,26], show that both mammalian and leishmanial GDP-MPs have similar affinities for both Man-1-P and GTP. Nonetheless, some bacterial and trypanosomal GDP-MPs have shown K*_m_* values in the same range, from 8 to 15 µM for Man-1-P, but displayed lower affinity for the substrate GTP, with high K*_m_* values varying between 40 and 200 µM [21,30,32,33,34,35]. With V*_m_* of ≈0.6 µM·min^−1^ and k*_cat_* between 12.5 and 13 min^−1^, *Li*GDP-MP exhibited slightly higher velocities and turnover rates with both substrates in comparison to both *h*GDP-MP and *Ld*GDP-MP, with the latter displaying V*_m_* values between 0.30 and 0.39 µM·min^−1^ and k*_cat_* values between 6.23 and 8.05 min^−1^ (Table 1) [16]. Moreover, the catalytic efficiency (k*_cat_*/K*_m_* ratios) values of both *Li*GDP-MP and *Ld*GDP-MP were similar, between 0.5 and 0.85 min^−1^µM^−1^, and were slightly lower than those of *h*GDP-MP previously estimated to be between 1.24 and 1.87 min^−1^µM^−1^ [16,22]. Nevertheless, these k*_cat_* and k*_cat_*/K*_m_* values are markedly lower than those previously determined in some bacterial GDP-MPs, varying from 360 to 6480 min^−1^ for k*_cat_*, and from 9 to 90 min^−1^µM^−1^ for k*_cat_*/K*_m_* [36,37], indicating that the differences in velocities, turnover rates, and catalytic efficiencies observed between *Li*GDP-MP, *Ld*GDP-MP, and *h*GDP-MP are modest.

Moreover, the mechanism of reaction of *Li*GDP-MP was determined as a sequential random mechanism, where a ternary complex composed of GTP, Man-1-P, and the enzyme is formed. These data are in contrast with a previous work showing a ping-pong mechanism for a bacterial nucleotidyltransferase, where a product is released after the fixation of the first substrate but before the fixation of the second [38]. However, they are in agreement with several studies showing the formation of a ternary complex with a sequential mechanism for some leishmanial, human, or bacterial GDP-MPs, or other nucleotidyltransferases, such as ADP-glucose, TDP-glucose, and UDP-glucose pyrophosphorylases [16,21,39,40,41,42]. Furthermore, both leishmanial and human GDP-MPs and mammalian nucleotidyltransferase have been described to exhibit a sequential random mechanism [16,42], which is consistent with our data regarding *Li*GDP-MP.

Altogether, these results further highlight common biochemical and enzymatic properties between both *Li*GDP-MP and *Ld*GDP-MP. These leishmanial GDP-MPs have similar quaternary structure stability, substrate or cofactor specificities, K*_m_* and k*_cat_*/K*_m_* kinetic constants, and mechanisms of reaction. The only differences noticed between *Li*GDP-MP and *Ld*GDP-MP concerned the V*_m_* and k*_cat_* kinetic constants, but these differences were minor. Therefore, the single A258D mutation in *Li*GDP-MP did not substantially change the biochemical or enzymatic properties in comparison to *Ld*GDP-MP. This residue mutation may have changed other GDP-MP properties, such as the protein half-life or partners within the parasite, potentially providing an evolutionary advantage for *L. infantum* and *L. donovani*. Further biochemical and cellular studies are necessary to verify this hypothesis. Consequently, *h*GDP-MP displayed the same peculiarities in comparison to both *Li*GDP-MP and *Ld*GDP-MP: *h*GDP-MP is an enzyme composed of four subunits α and eight subunits β displaying a slightly more acidic optimal pH, a lower Mg^2+^ optimal concentration, a weak but substantial enzyme activity in the presence of ITP, and moderately higher affinities and catalytic efficiencies for both Man-1-P and GTP in comparison to both leishmanial GDP-MPs. These differences regarding the biochemical and enzymatic properties between human and leishmanial GDP-MPs could be further exploited to design new GDP-MP inhibitors. Especially, a comparison of the quaternary structures of leishmanial and human GDP-MPs, which are homo-hexameric and hetero-dodecameric, respectively, is a promising approach to the design of new antileishmanial compounds that specifically inhibit the enzyme of this parasite.

## Figures and Tables

**Table 1:** Kinetic constants (V*_m_*, K*_m_*, k*_cat_*, k*_cat_*/K*_m_*) of *Li*GDP-MP, *Ld*GDP-MP, and *h*GDP-MP for both substrates Man-1-P and GTP.

	Substrate	V*_m_* (µM·min^−1^) ± SD	K*_m_* (µM) ± SD	k*_cat_* (min^−1^) ± SD	k*_cat_*/K*_m_* (min^−1^µM^−1^) ± SD	Reference
*Li*GDP-MP	Man-1-P	0.60 ± 0.02	14.72 ± 1.45	12.47 ± 0.47	0.85 ± 0.06	This study
	GTP	0.63 ± 0.02	17.95 ± 1.61	13.13 ± 0.43	0.73 ± 0.04	
*Ld*GDP-MP	Man-1-P	0.30 ± 0.06	9.98 ± 6.15	6.23 ± 1.24	0.75 ± 0.33	[14]
	GTP	0.39 ± 0.03	16.84 ± 3.29	8.05 ± 0.71	0.48 ± 0.05	
*h*GDP-MP	Man-1-P	0.47 ± 0.01	6.98 ± 0.64	9.30 ± 0.19	1.34 ± 0.11	[14]
	GTP	0.44 ± 0.01	7.08 ± 0.57	8.74 ± 0.14	1.24 ± 0.10	

## Data Availability

Not applicable.

## Supplementary Materials

The following supporting information can be downloaded at: https://www.mdpi.com/article/10.3390/microorganisms10020231/s1, Table S1: Optimal parameters of *Li*GDP-MP, *Ld*GDP-MP and *h*GDP-MP enzymatic reactions; Figure S1: Mass spectrometry analysis of *Li*GDP-MP; Figure S2: Determination of optimal reaction time of *Li*GDP-MP; Figure S3: Determination of *Li*GDP-MP optimal concentration of enzyme assay; Figure S4: Determination of *Li*GDP-MP optimal conditions of enzymatic reaction; Figure S5: Determination of *Li*GDP-MP kinetic constants for both substrates GTP and Man-1-P.
