# Supplementary material for: Minor Impact of A258D Mutation on Biochemical and Enzymatic Properties of Leishmania infantum GDP-Mannose Pyrophosphorylase
Source: Microorganisms. 2022 Jan 21;10(2):231. doi: 10.3390/microorganisms10020231 (PMC8877407; doi:10.3390/microorganisms10020231)

Supplementary Materials

Table S1. Optimal parameters of *Li*GDP-MP, *Ld*GDP-MP and *h*GDP-MP enzymatic reactions. All enzymatic reactions were performed with a reaction time of 20 min.

	[GDP-MP] (ng/µL)	[GTP] (µM)	[Man-1-P] (µM)	Temperature (°C)	pH	[Mg^{2+}]	Reference
*Li*GDP-MP	2	100	100	37	7.6	5	This study
*Ld*GDP-MP	2	100	100	37	7.6	5	[14]
*h*GDP-MP	2	100	100	37	6.5	1	[14]

1 MSASDGQGMR AVILVGGFGT RLRPLTLTTP KPLVPFCNKP MIIHQIEALK
51 AVGVTEVILA VAYRPEAMKE QMDEWSRKLG VSFVFSVEEE PLGTAGPLAL
101 ARDILMQDDK PFFVLNSDVT CTFPMQELLD FHKAHGGEGT IMVSQVTQWE
151 KYGVVVYSPQ NYQIERFVEK PSRFLGDRIN AGIYIFNKSI LDRIPPRRTS
201 IEKEIFPAMA AEGQLYAFNL EGFWMDVGQP KDYILGMTKF IPSLVHGNRE
251 TEQLHTEDME HQRGGRFTVI GASLIDPSAK IGDGAVIGPY ASIGANCVIG
301 ESCRIDNAAI LENSKVGKGT MVSRSIVGWN NRIGSWCHIK DISVLGDDVE
351 VKDGVILIGT KVLPNKDVGE HRFEPGIIM

Figure S1. Mass spectrometry analysis of *Li*GDP-MP. MALDI-TOF peptide fingerprints of the purified *Li*GDP-MP. The peptide identified are highlighted in red. The protein sequence coverage is 73%.

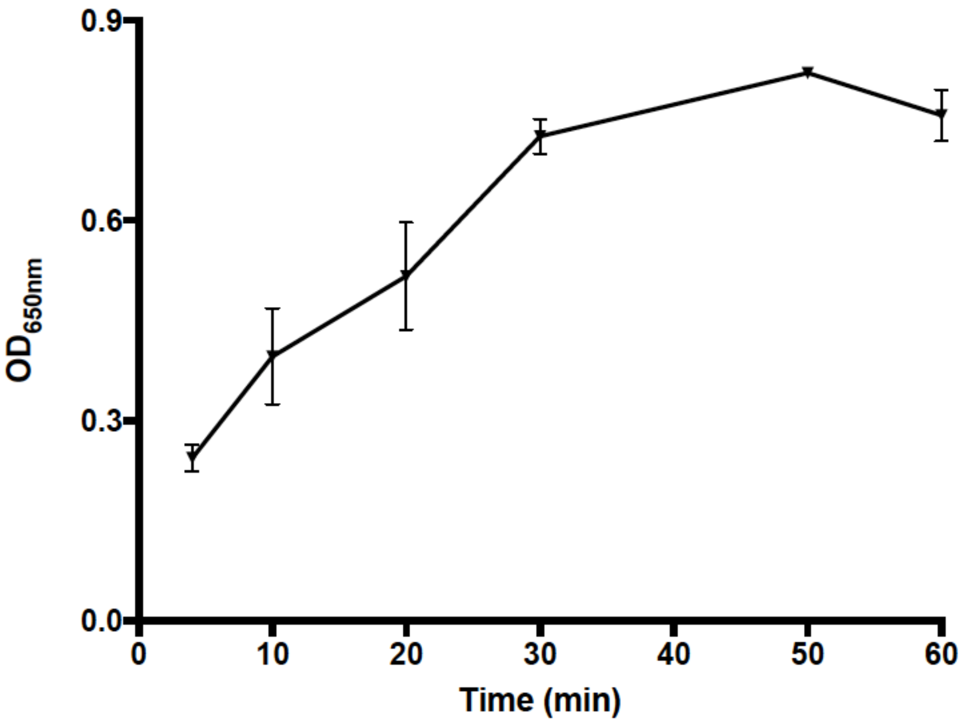

Figure S2. Determination of optimal reaction time of *Li*GDP-MP. The OD_{650nm} was plotted as a function of time of reaction. All reactions were performed at optimal temperature, pH, Mg^{2+}, enzyme and substrates concentrations. The results expressed correspond to the mean of three independent experiments ± SD.

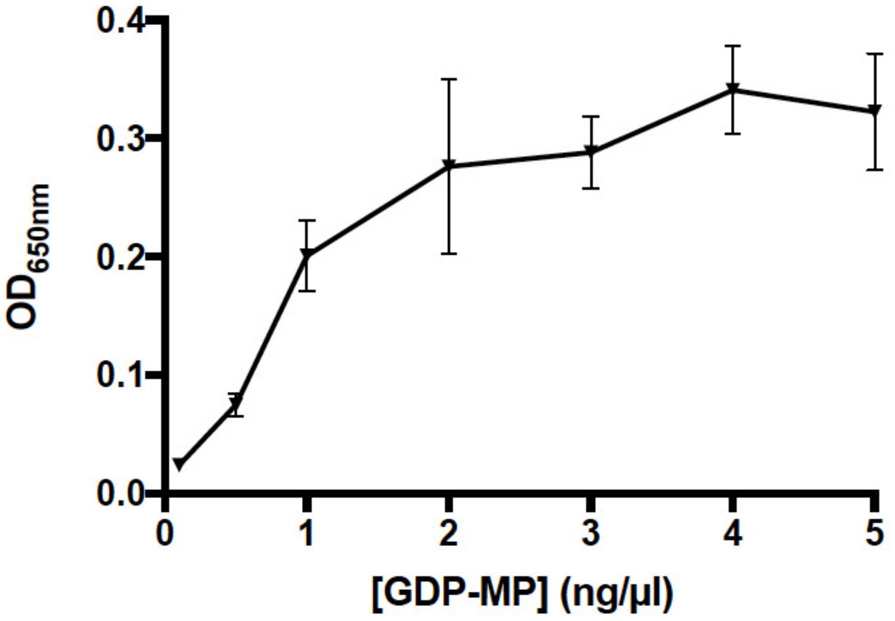

Figure S3. Determination of *Li*GDP-MP optimal concentration for enzyme assay. The OD_{650nm} was plotted as a function of *Li*GDP-MP concentration. All reactions were performed at optimal time, temperature, pH, Mg^{2+} and substrate concentrations. The results expressed correspond to the mean of three independent experiments ± SD.

Figure S4. Determination of *Li*GDP-MP optimal conditions of enzymatic reaction. Activity of *Li*GDP-MP as a function of temperature (**A**), pH (**B**), Mg^{2+} (**C**), GTP (**D**) or Man-1-P (**E**) concentrations. For the determination of the optimal pH, the buffers used are 50 mM MES for pH 5.6 and 6.5, and 50 mM Tris-HCl for pH between 6.8 and 10. (D) Michaelis-Menten plot V = f([GTP]) where Man-1-P concentration was held constant at 150 µM. (E) Michaelis-Menten plot V = f([Man-1-P]) where GTP concentration was held constant at 150 µM. In each graph, enzyme activities were calculated relative to the maximal activity obtained and are expressed in percent. The results correspond to the mean of three independent experiments ± SD.

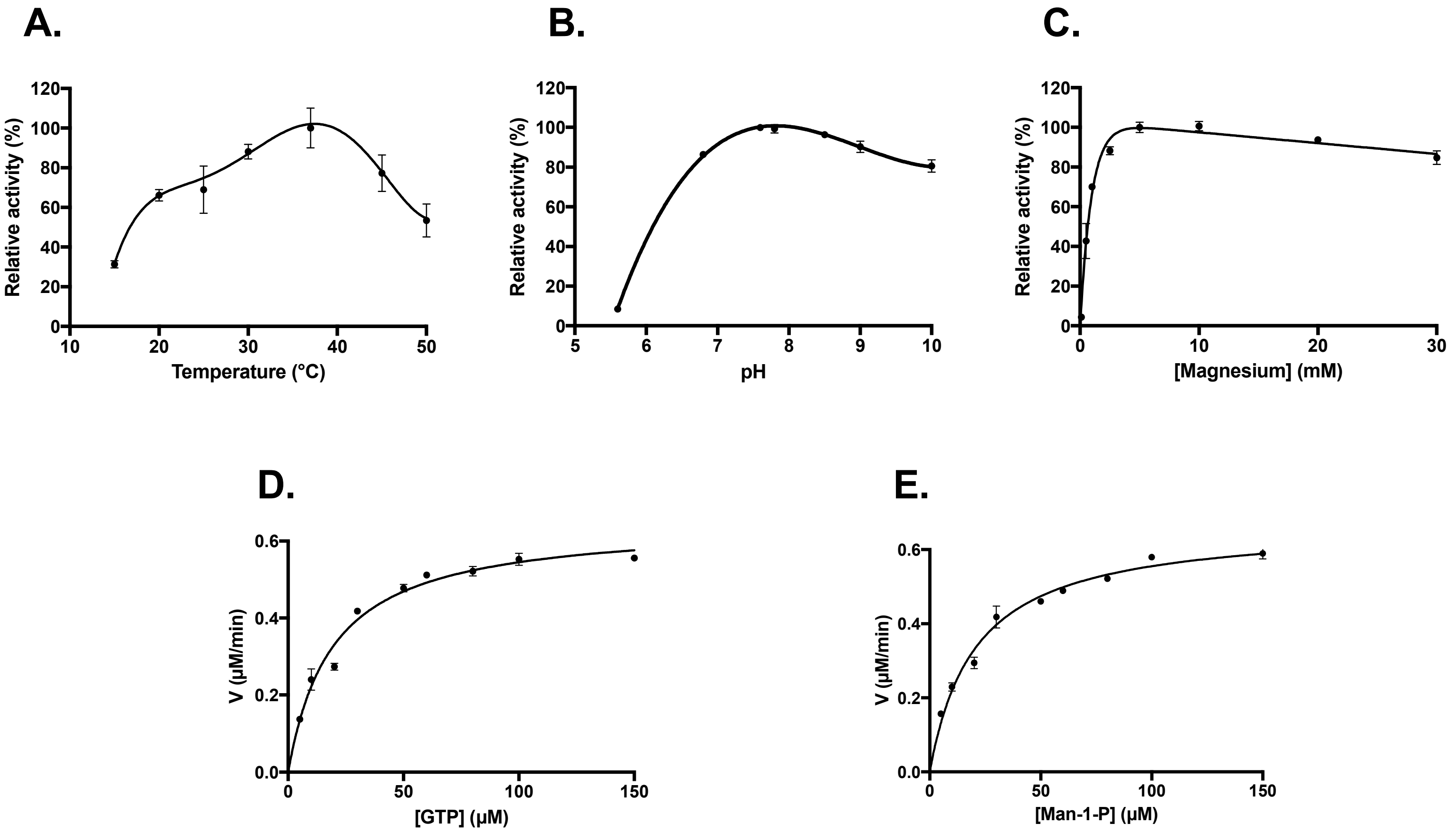

Figure S5. Determination of *Li*GDP-MP kinetic constants for both substrates GTP and Man-1-P. (**A**) Lineweaver Burk double reciprocal plot 1/V = f(1/[GTP]). Man-1-P concentration was held constant at 150 μM. (**B**) Lineweaver Burk double reciprocal plot 1/V = f(1/[Man-1-P]). GTP concentration was held constant at 150 μM. The results expressed correspond to the mean of three independent experiments ± SD.

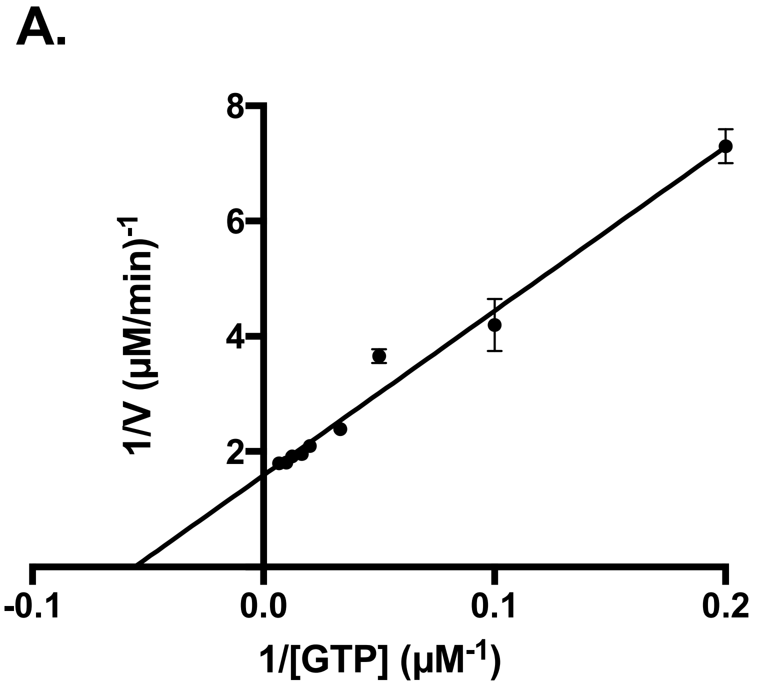

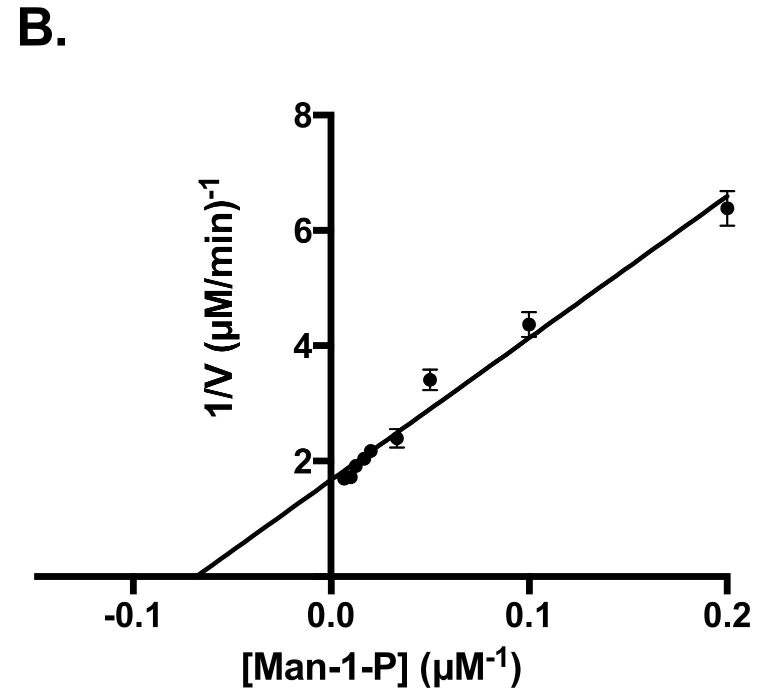

Supplement: Supplementary file 1 [file microorganisms-10-00231-s001.zip › microorganisms-1559561-supplementary.pdf]
